# An Archaeometric Characterization of Ecuadorian Pottery

**DOI:** 10.1038/s41598-018-38293-w

**Published:** 2019-02-25

**Authors:** Alejandra Sánchez-Polo, Sarah Briceño, Alex Jamett, Salomé Galeas, Orlando Campaña, Víctor Guerrero, Carlos R. Arroyo, Alexis Debut, Duncan J. Mowbray, Camilo Zamora-Ledezma, Jorge Serrano

**Affiliations:** 1 Yachay Tech University, School of Physical Sciences and Nanotechnology, 100115 Urcuqui, Ecuador; 2 Museo de Arte Precolombino - Casa del Alabado, Quito, Ecuador; 3 Instituto Venezolano de Investigaciones Científicas (IVIC), Apartado 20632, Caracas, 1020-A Venezuela; 4 Escuela Politécnica Nacional, Departamento de Materiales, Quito, Ecuador; 5 Centro de Nanociencia y Nanotecnología, Universidad de las Fuerzas Armadas ESPE, Sangolqui, Ecuador; 6 Departamento de Prehistoria, Historia Antigua y Arqueología, Universidad de Salamanca, Salamanca, Spain

## Abstract

Ecuadorian pottery is renowned for its beauty and the particularly rich colour of its pigments. However, a major challenge for art historians is the proper assessment of the provenance of individual pieces due to their lack of archaeological context. Of particular interest is the Jama-Coaque culture, which produced fascinating anthropomorphic and zoomorphic pottery from ca. 240 B.C. until the Spanish Conquest of 1532 A.D. in the coastal region of Ecuador. Using a combination of microscopic and spectroscopic techniques, i.e., transmission electron microscopy (TEM), Raman spectroscopy, Fourier transform infrared spectroscopy (FTIR), energy-dispersive x-ray spectroscopy (EDX), and scanning electron microscopy (SEM); we are able to characterize these pieces. We have found several kinds of iron-oxide based nanostructures in all the colour pigments we investigated for the Jama-Coaque culture, suggesting the same unique volcanic source material was used for their clay. Such nanostructures were absent from the pigment samples studied from other contemporary coastal-Ecuadorian cultures, i.e., the Tumaco-La Tolita and Bahía cultures. In the yellow pigments of goethite we find carbon nanofibres, indicating these pigments were subjected to a thermal treatment. Finally, in the blue, green, and black pigments we detect modern pigments (phthalocyanine blue, lithopone, and titanium white), suggesting modern restoration. Our results demonstrate the power of TEM, Raman, FTIR, EDX, and SEM archaeometric techniques for characterizing pieces without a clear archaeological context. Furthermore, the characterization of nanostructures present in such pieces could be used as a possible fingerprint for a provenance study.

## Introduction

In the 21st century, the interpretation, analysis, and understanding of ancient works of art requires an interdisciplinary approach involving art historians, archaeologists, chemists, spectroscopists and material scientists^1–3^. Pottery was one of the main productions of the pre-Columbian cultures in South America. Their study can provide new insight into and understanding of the social development, political system, and technology available at that time.

In the case of Ecuador, however, most of the pottery available stems from site looting. The lack of an exact determination of their archaeological sites and geological context^4^ poses a challenge for modelling the evolution of these cultures in a conclusive manner and determining the provenance of these pieces. Studies of the geological sites provide one way to access this information^5^. Another way is to study the structural and chemical properties of ceramics and other artifacts - also referred to as archaeometry research. The quality and preservation of pigments in their ceramics, textiles and paintings is a signature of their level of technology. Recent advances in non-invasive instrumentation have opened a window on these ancient potter’s choices of materials, mastery of their processing and application, and the society in which they lived^6,7^.

**Figure 1:**
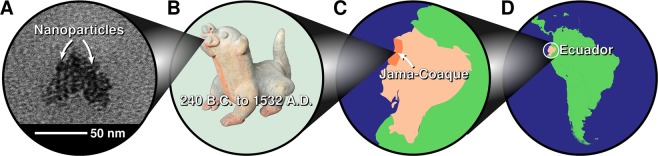
(**A**) TEM micrograph of nanoparticles present in (**B**) pigment on pottery from (**C**) the Jama-Coaque culture of (**D**) South America.

Here we apply state-of-the-art spectroscopic and microscopic techniques to characterize pottery from the Jama-Coaque culture of Ecuador (240 B.C. to 1532A.D.)^5,8–12^ which occupied 250 km of coastline between the Bahía de Caráquez and the Cabo de San Francisco (Fig. 1)^8,9^. These pigments display an extraordinary state of preservation as compared to those of contemporary Ecuadorian cultures, such as the Tumaco-La Tolita and Bahía.

This subject has been widely treated in Mexico and Peru, where a variety of pigment sources have been identified – from mineral (hematite in red pigment), animal (cochinilla in carmine pigment), and vegetal (indigo in Mayan blue) origins^13^. The application of pigments and dyes has been studied in murals, textiles and figures^14^. However, in South America, restorations, e.g., repaintings, of ancient antiquities have been driven by the insatiable demand of western antiquity markets. Moreover, pastiches of broken original pottery are often designed to appeal to the eye of a western collector and hence demand higher prices^15,16^. Using archaeometry, we can differentiate between original, restored, and altered aspects within the same piece^17^. For example, with these techniques we can identify modern chemical compounds, or find nanostructures to identify common sources of materials.

**Figure 2:**
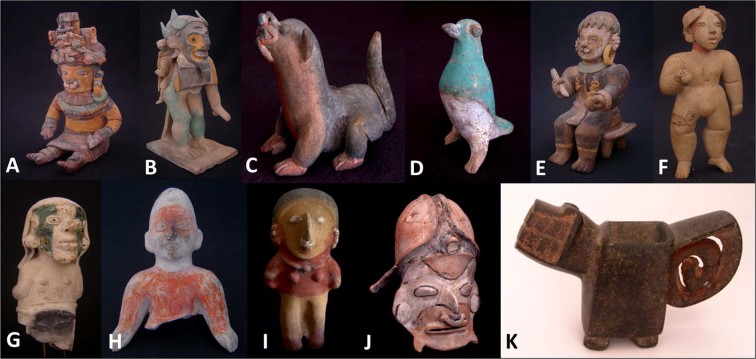
Representative coloured anthropomorphic and zoomorphic pottery of (**A–F**) Jama-Coaque, (**G**,**H**) Tumaco-La Tolita, (**I**,**J**) Bahía (**K**), and Valdivia cultures.

Here we have studied 11 representative coloured anthropomorphic and zoomorphic pieces of Jama-Coaque (A–F), Tumaco-La Tolita (G,H), Bahía (I, J), and Valdivia (K) coastal-Ecuadorian cultures, shown in Fig. 2, with the aim of identifying similarities and differences between contemporary pottery, in particular in the use of colour and pigments. All these pieces are part of the collection of the Museum of Pre-Columbian Art Casa del Alabado in Quito, Ecuador^18^. The quality of Jama-Coaque ceramics, both in colour and in preservation, is obviously excellent at first sight^19^. Some influences from northern Mesoamerican cultures may be inferred from the use of post baking painting over anthropomorphic, animal, and mixed anthropomorphic and zoomorphic figures of the Tumaco-La Tolita, Jama-Coaque and Bahía cultures^20^. Despite the variety of figures and the applied paintwork, only a few archaeometric studies of Ecuadorian cultures have focused on their pigments^8,21^.

## Results

**Figure 3:**
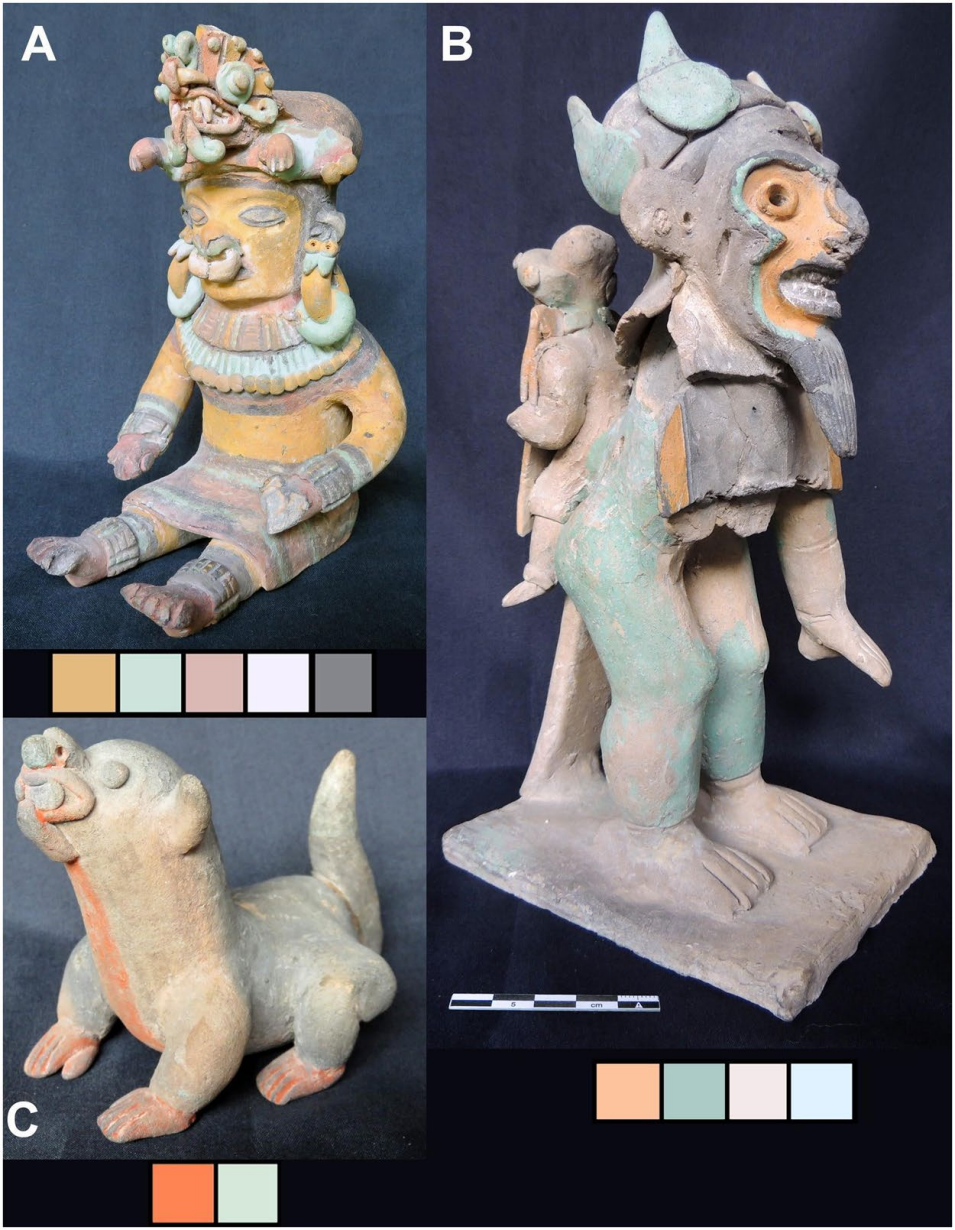
Selected Jama-Coaque pottery samples (**A**–**C**) with colour palettes. All photographs are displayed with a similar scale and a palette showing all sampled colours.

Figure 3 displays photographs of three samples of the Jama-Coaque collection we investigated, designated hereafter as samples **A**, **B**, and **C**. Below each photograph, the palette of colours investigated for each sample is displayed for reference. The contrast in colour and craft with pottery stemming from other contemporary Ecuadorian cultures can be appreciated from Fig. 2. Among cultures of the Americas, Jama-Coaque potters are well-known for their lavish use of colour, both in range and variety^8,19,22^.

Micrometer size extractions were obtained from each of the colours present on the pieces and investigated using TEM, Raman and FTIR spectroscopy, and EDX/SEM. We will focus hereafter on the findings we obtained by applying these techniques to samples **A**, **B**, and **C** from the Jama-Coaque collection.

### TEM

**Figure 4:**
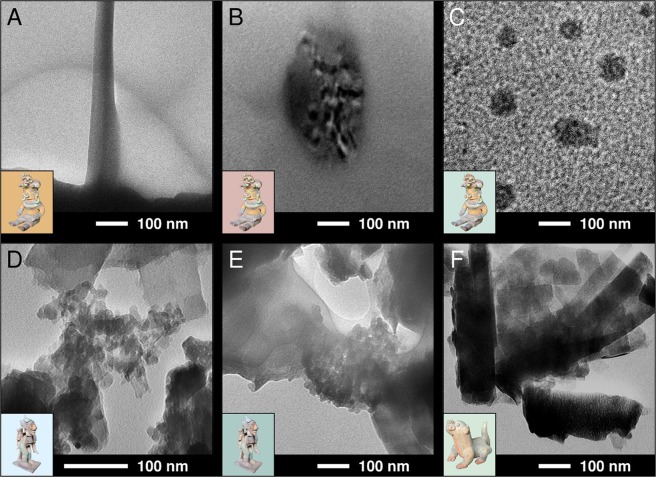
TEM micrographs obtained from the pigments of the pieces shown in Fig. 3, with the sample and colour as background shown as insets.

**Figure 5:**
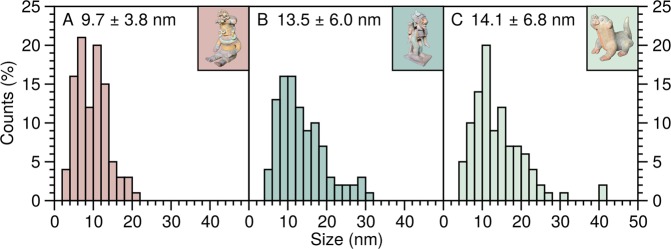
Histogram and average nanoparticle size for the pigments from the pieces shown in Fig. 3, with the sample and colour as background shown as insets.

Red and yellow pigments are typically associated with iron-oxide related minerals, specifically, hematite and goethite^23,24^. It is well known that, under heating, goethite dehydrates forming hematite, which has been shown to be initially formed as micro or nano domains. These domains coalesce at higher temperatures leading to a more crystalline compound^25^. The results of the nucleation and domain growth process, i.e., carbon nanofibres, are observed in the TEM micrograph shown in Fig. 4A, which corresponds to the yellow pigment of sample **A** (Fig. 3A). This sample also contains metallic nanoparticles within nanovesicles (Fig. 4B) and naked nanoparticles (Fig. 4C) in the extractions corresponding to red and green pigments, respectively. We have found a mixture of nanoparticles with nanoribbons in the blue pigment of sample **B**, as shown in Fig. 4D. The presence of organic binding and embedding nanoparticles is observed as a veil with different contrast in Fig. 4E, which corresponds to a TEM micrograph obtained from the green pigment of this statue. Micrographs of a similar colour present in sample **C** (Fig. 3C) display nanosticks, as observed in Fig. 4F. These structures may correspond to hematite^26^. Most importantly, the presence of nanoparticles and different nanostructures, with an average particle size of 12.4 ± 6.0 nm, is a feature found in all of the pigments we have examined from the Jama-Coaque culture. Histograms of the nanoparticle size distribution for pigments from samples **A**, **B**, **C** are shown in Fig. 5.

### Raman

Raman spectroscopy has been widely used to identify pigments, both natural and synthetic, in ancient pottery, figures and painting^27^. Furthermore, the combination of this technique with infrared spectroscopy allows one to ascertain the organic or inorganic origin of pigments, as well as to detect the presence of binders and different kinds of clay employed in the manufacturing process^28–32^. This analysis is challenging for the archaeological pieces analyzed herein due to our lack of information about the specific sites where they were originally found.

**Figure 6:**
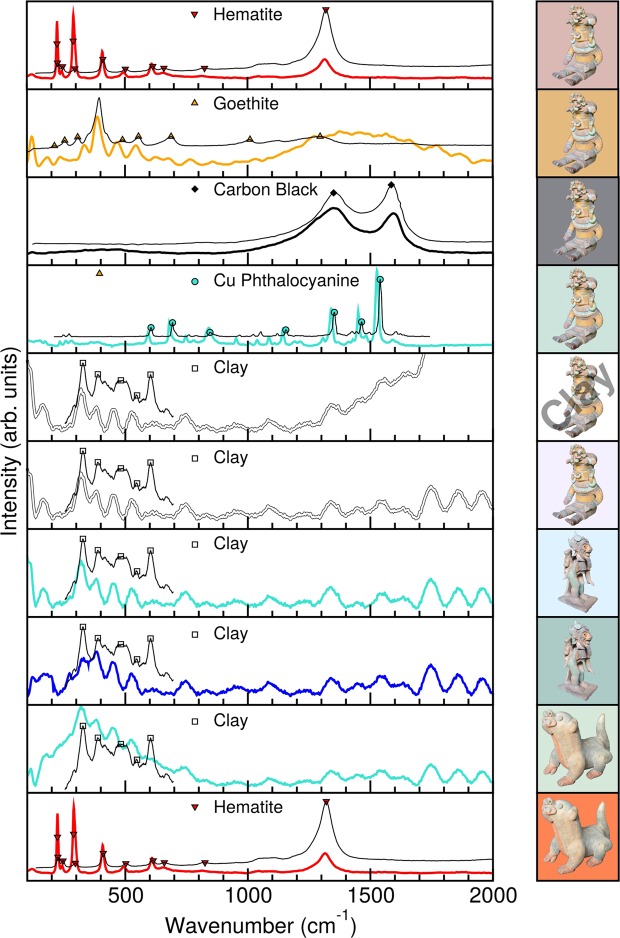
Raman spectra (λ_exc_ = 633 nm) for selected pigments present in Jama-Coaque ceramics (thick lines). Reference spectra (thin lines) and selected peaks for clay (squares)^39^, copper phthalocyanine (circles^)37^, goethite (triangle up)^34^, hematite (triangle down)^33^, and carbon black (diamond)^35^ are provided for comparison. The sample and colour as background are provided to the right of each spectra.

Figure 6 shows selected typical Raman spectra for the pigmented areas obtained from the Jama-Coaque pieces of Fig. 3, with the positions of the main vibrational modes provided in Table 1. In the red pigments of samples **A** and **C** we identified the presence of iron oxide in the hematite phase, *α*-Fe_2_O_3_^25,29,33^. In a similar way, the yellow pigment of sample **A** exhibits a very intense band in its Raman spectrum at 398 cm^−1^, corresponding to the goethite phase of iron oxide, α-FeOOH^34^. We also notice other broad peaks that can be related to heat treatment and a potential phase transformation as a function of the temperature, as reported previously^29,31^. In the black pigment of sample **A** we recognize typical carbon-related features. In our case, the *sp*^3^ (D band) is slightly stronger than in carbon black^35^, suggesting a higher density of defects^29,36^. The green pigment of sample **A** exhibits very intense bands at 1352, 1465, and 1539 cm^−1^ which we assign to copper phthalocyanine^37,38^. We have also performed a Raman study on samples of bare clay for sample **A**, where we found two broad peaks at 327 and 388 cm^−1^, which are typical features of heat treated clay^39^. However, a similar Raman spectra is observed for the white pigment of sample **A**, the green and blue pigments of sample **B**, and the green of sample **C**, indicating these extracted samples were mainly composed of clay. To both clarify these issues and verify our findings we also performed measurements on all the samples using the complementary FTIR technique.

**Table 1:** Raman vibrational modes from Fig. 6 and their assignments.

Sample	Raman (cm^−1^)	Assignment
Black	1350, 1585, 2700, 2900	Carbon Black^a,b,c^
Yellow	230, 398, 470, 540	Goethite^d^
Red	290, 550, 605, 670, 1315	Hematite^e^
Green	606, 694, 850, 1155, 1352, 1465, 1539	CuPc^e^
Clay	327, 388, 482, 548, 603	Clay^f^

^a^ref.^23^. ^b^ref.^49^. ^c^ref.^35^. ^d^ref.^20^. ^e^ref.^37^. ^f^ref.^39^.

### FTIR

**Figure 7:**
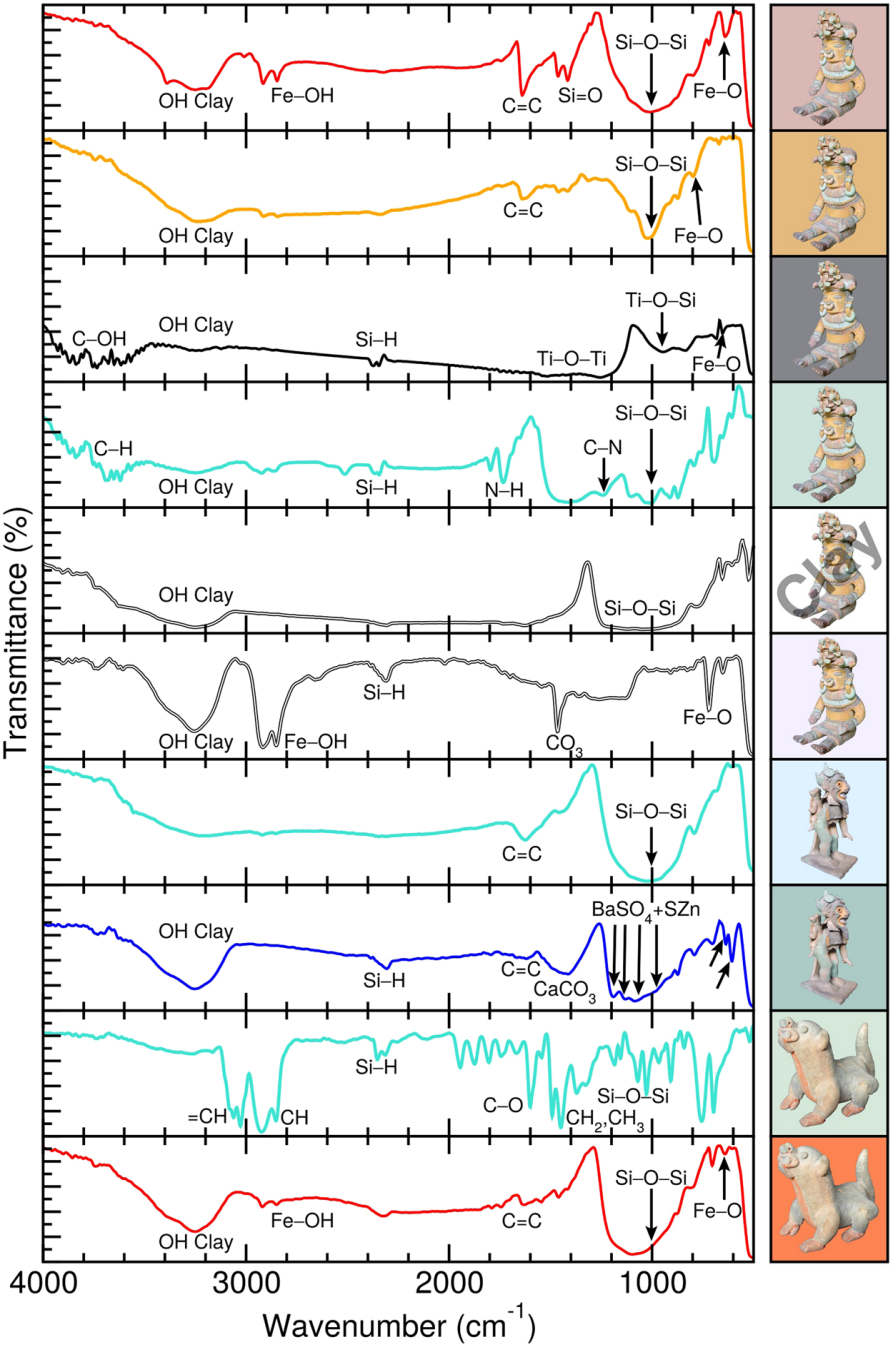
Fourier transform infrared (FTIR) spectra for selected pigments present in Jama-Coaque pottery. The sample and colour as background are provided next to each spectrum.

**Figure 8:**
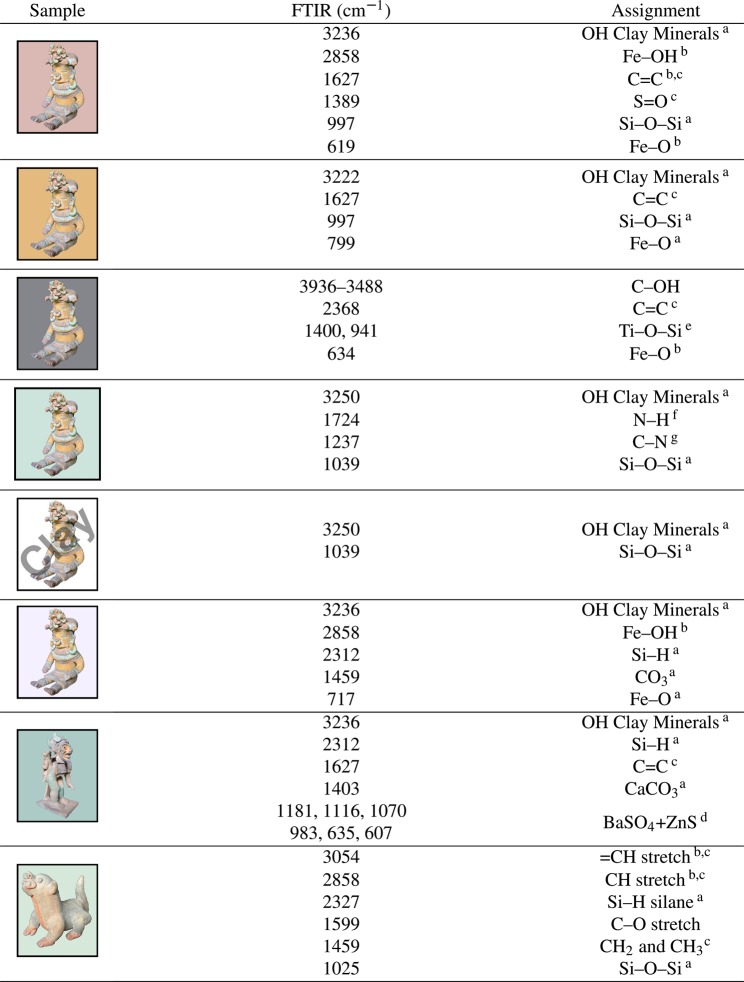
Fourier transform infrared (FTIR) absorption peaks from Fig. 7 and their assignments. ^a^ref.^23^. ^b^ref.^50^. ^c^ref.^41^. ^d^ref.^45^. ^e^ref.^44^. ^f^ref.^51^. ^g^ref.^42^.

We have performed FTIR measurements (Fig. 7) for the same selected pigments we have studied using Raman spectroscopy (Fig. 6). The positions of the main FTIR absorption peaks and their assignment is provided in Fig. 8. In Fig. 7 the FTIR absorption between 3236 cm^−1^ and 3250 cm^−1^ is due to hydroxyl ions of the clay minerals, and the peak at 3140 cm^−1^ can be attributed to hydrated ferric oxide. These peaks allow us to distinguish between hematite and goethite samples^23,25,40^. The yellow pigment’s infrared spectrum was found to contain many similarities with reported goethite mineral spectra^23^. The stretching vibrations of calcium carbonate at 1403, 872, 705, and 611 cm^−1^ were identified in the blue samples^23^. Amorphous silica was also present in the samples (Fig. 8)^23^. Peaks observed at 1450 and 1025 cm^−1^ are assigned to the Si–O bond stretching, while weak peaks at 694 and 776 cm^−1^ and the shoulder at about 521 cm^−1^ are assigned to deformation modes involving the Si-O moiety and attributable to quartz SiO_2_^23^. The very weak absorption peaks at 1627 cm^−1^ corresponding to the C=C group suggest the presence of organic materials in the samples used as binders^41^. The absorption peaks seen at 1237 and 1737 cm^−1^ for the green pigment of sample **A** are consistent with the C–N and N–H stretching modes of copper phthalocyanine^42,43^. Altogether, these FTIR results are consistent with our findings from Raman spectroscopy.

For the black pigment of sample **A**, we found absorption peaks at 941 and 1400 cm^−1^, which are consistent with the presence of titanium dioxide^44^. For the blue pigment of sample **B** we found six absorption peaks which are the fingerprint of BaSO_4_ + ZnS, i.e., lithopone, shown in Fig. 7^45^. To verify the presence of these pigments in our samples, we have performed a complementary elemental analysis.

### EDX/SEM

**Figure 9:**
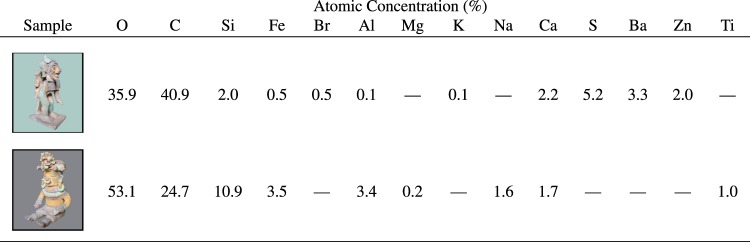
Energy-dispersive x-ray spectroscopy (EDX) elemental analysis.

The EDX/SEM elemental analysis for the black pigment of sample **A** and the blue pigment of sample **B** are provided in Fig. 9. We found the black pigment of sample **A** contained 1% Ti, consistent with a whitening by titanium dioxide of the carbon black pigment found using Raman spectroscopy (Fig. 6). Surprisingly, the EDX/SEM measurement of the blue pigment from sample **B** several percent S, Ba, and Zn in approximately a 2:1:1 ratio, consistent with the chemical composition of lithopone.

## Discussion

**Figure 10:**
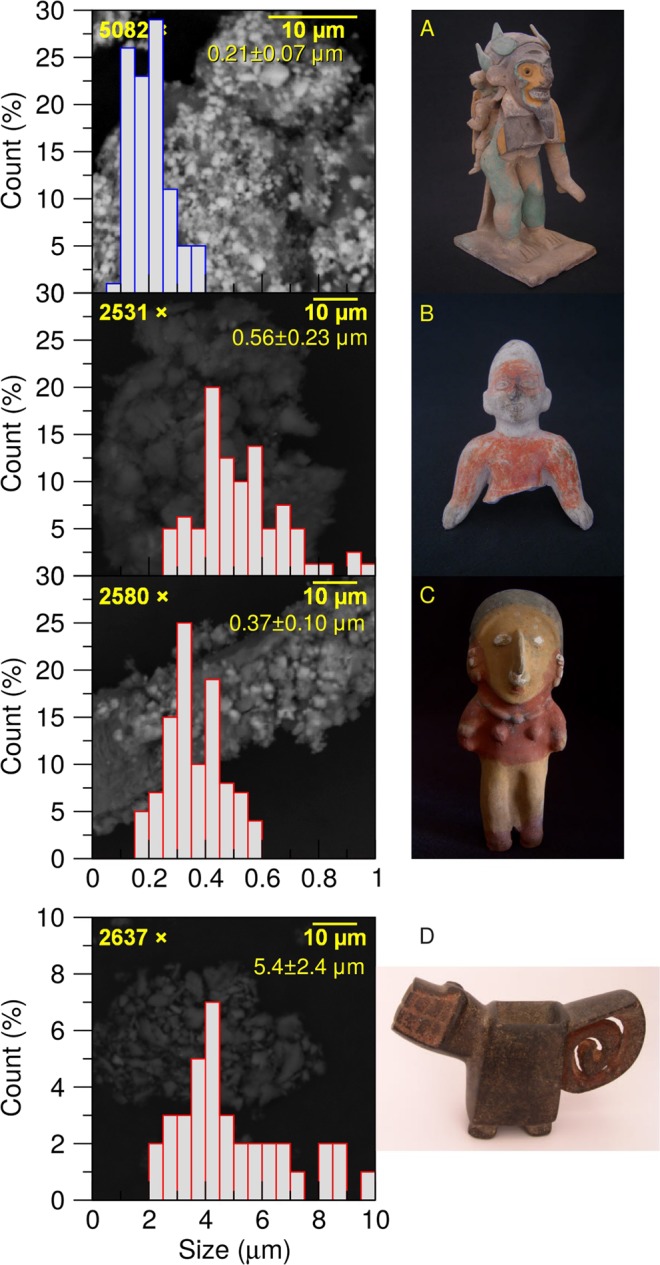
Histogram and average particle size of scanning electron migrographs (shown as background) for pigments from (**A**) Jama-Coaque, (**B**) Tumaco-La Tolita, (**C**) Bahía, and (**D**) Valdivia cultures.

To the best of our knowledge, this is the first time evidence has been found of nanostructures in painted pottery from coastal-Ecuadorian cultures. Using TEM, we have found several different types of nanostructures, e.g., carbon nanofibres, nanotubules, and nanovesicles. Surprisingly, we found iron-oxide based nanostructures in all the colour pigments we investigated for the Jama-Coaque culture. This strongly suggests that the same source material was used for their clay. It is very important to highlight that such nanostructures were absent from the pigment samples studied from other coastal-Ecuadorian cultures, i.e., the contemporary Tumaco-La Tolita and Bahía cultures, and the earlier Valdivia culture, as seen in the SEM images of Fig. 10.

The presence of naturally occurring nanomaterials in the environment, from inorganic ash, soot, iron, and mineral particles found in the air or in soils, is well known^46^. One of the sources of the nanomaterials found in the clay and pigments used by the Jama-Coaque culture could be related with the volcanic eruption in ∼90 A.D^5^. and the presence of iron oxide nanoparticles in the resulting volcanic ash. Altogether, this suggests that the pottery from which samples **A**, **B**, and **C** are composed is (1) from the same source, (2) from the same period, (3) unique to the Jama-Coaque region, and (4) most probably “original”.

Metallic nanoparticles act as a catalytic nucleus for nanofibre formation at high temperatures (≳500 °C). Their presence in the yellow goethite pigment of sample **A** indicates the pigment underwent a heat treatment. Using TEM and FTIR we have confirmed the presence of organic materials in the pigments related with the use of binders^23^ combining with the inorganic iron oxide sources for the preparation of the pigments. In fact, the goethite and hematite pigments of sample **A** are quite similar in colour to the Jama-Coaque statue from the Metropolitan Museum of Art shown in ref.^14^. This suggests the goethite and hematite pigments of samples **A** and **C** could be “original”.

It is worth mentioning that we have also found different synthetic compounds, i.e., copper phthalocyanine (CuPc) in the green of sample **A**, BaSO_4_ + ZnS in the blue of sample **B** and titanium dioxide in the black of sample **A**, via a combination of spectroscopic and microscopic techniques. Note that CuPc, i.e., phthalocyanine blue, was discovered in 1929^37,38^, while BaSO_4_ + ZnS, i.e., lithopone, was discovered in the 1890s by DuPont^47^. These findings show that samples **A** and **B** have been either restored or partially altered from their “original” condition.

The use of modern pigments to either restore or alter original pottery is a common practice for obtaining higher prices in western antiquities market. Our results are consistent with the unusual characteristics of sample **B** compared to typical Jama-Coaque ceramics, which suggest this piece is a pastiche, i.e., a combination of several broken original pieces^5,17,22^. Sample **B**, as shown in Fig. 3B, has a rather haphazardly attached head and an unusual back-brace or “pillar”. More surprisingly, the main facial features of sample **B** do not conform to the typical Jama-Coaque stylistic canon^5,17,22^.

## Conclusions

Our results show how spectroscopic and microscopic techniques, such as TEM, Raman, FTIR, and EDX/SEM, may be used to characterize Jama-Coaque pigments and pottery. We have found several different types of iron-oxide based nanostructures in all the colour pigments we investigated from the Jama-Coaque culture, suggesting the same volcanic source material was used for their clay. Such nanostructures were absent from the pigment samples studied from other contemporary coastal-Ecuadorian cultures, i.e., the Tumaco-La Tolita and Bahía cultures. In the yellow pigments of goethite we find carbon nanofibres, indicating these pigments were subjected to a thermal treatment. Unexpectedly, in the blue, green, and black pigments we detect modern pigments (phthalocyanine blue, lithopone, and titanium white). Using archaeometric techniques we have been able to distinguish between various restoration treatments which have been applied to the pigments used in these pieces. Additionally, this has provided us the opportunity to evaluate and verify the painting techniques used and origin of the sources employed to prepare the pigments. This work opens the door to further research on a larger collection of Ecuadorian pottery to demonstrate the significance of the nanostructures revealed here as a possible fingerprint for future provenance studies.

## Methods

### Sample description

In order to determine the structural and chemical composition of the pigments, micrometer size samples were carefully extracted using a scalpel with a blade that was changed after each extraction. Samples were scraped with the tip of the scalpel obtaining grains of 0.1 mm^2^, and stored in a clean/sterile vessel. Samples were collected at the Casa del Alabado Museum in Quito for each of the colours present on the pieces. To determine whether there was any variation over the samples, various areas of the samples were examined.

### Characterization

To characterize the samples, each pigment was investigated using transmission electron microscopy (TEM), Raman spectroscopy, Fourier transform infrared spectroscopy (FTIR), energy-dispersive x-ray spectroscopy (EDX), and scanning electron microscopy (SEM).

For TEM measurements, the samples were diluted in ethanol (99.99% purity, J.T.Baker). A drop of the solution was deposited on a formvar-carbon coated 300-mesh. The TEM micrographs were obtained at 80 kV by using a FEI-Tecnai G20 Spirit Twin microscope equipped with a Eagle 4k HR camera.

Raman spectra were collected at room temperature using a Horiba LabRAM HR evolution Raman spectrometer coupled with a CCD camera and excited by a solid-state laser (λ_exc_ = 633 nm). A microscope with a 100× objective lens (laser spot size of about 0.5 μm) was used to focus on the sample and its resolution was set to 0.35 cm^−1^. The power density was kept below 50 mW in order to avoid heating effects. The Raman spectrometer was calibrated using the 520 cm^−1^ line of a Si wafer. Spectra were recorded in the 100 to 2000 cm^−1^ region with a 1800 g/mm grating. The recording time was between 10 and 1000 s, and two accumulations per spectrum segment were averaged. All raw Raman spectra were systematically baseline corrected.

FTIR Infrared spectra were recorded using a Perkin Elmer 100 Series FT-IR spectrometer between 4000 and 500 cm^−1^. A gold substrate was used as a reflecting material.

EDX and SEM measurements were carried out using a Phenom ProX desktop SEM/EDS. The pigment samples were attached using conductive double-faced tape to the sample holder and observed without any further metallisation under standard conditions.

### Statistical analysis

Our statistical analysis of the nanoparticle size distribution was performed using the ImageJ software^48^. We measured nanoparticle sizes from TEM micrographs until we obtained a sample size of 100 measurements for each colour of pigment.

## Data Availability

The datasets generated during and/or analysed during the current study are available from the corresponding author on reasonable request.
